# High Death Anxiety and Ambiguous Loss: Lessons Learned From Teaching Through the COVID-19 Pandemic

**DOI:** 10.1093/geroni/igab046.295

**Published:** 2021-12-17

**Authors:** Raven Weaver, Cory Bolkan, Autumn Decker

**Affiliations:** 1 Washington State University, Pullman, Washington, United States; 2 Washington State University, Vancouver, Washington, United States

## Abstract

For gerontological educators, topics such as mortality, loss, and end-of-life issues often emerge or are central in their courses. However, the COVID-19 pandemic has raised our awareness of loss and death on a global scale and teaching during the pandemic has raised questions about how educators, communities, or systems of higher education can support students’ learning while simultaneously experiencing losses during intense times of uncertainty. In this mixed-method study of 246 students enrolled in undergraduate thanatology courses, we explored their levels of death anxiety and their experiences with pandemic-related losses. We found that students’ death anxiety increased significantly during the pandemic, in comparison to the years prior (p < .001). We also conducted a content analysis in a subset of students’ written narratives (n = 44) regarding their pandemic experiences. We identified three themes. Participants desired: (a) more flexibility from instructors, no questions asked; (b) more compassion and understanding; and (c) specific, targeted support resources. The voices of students were filtered through the authors’ interpretation as educators to provide several teaching recommendations that support student learning during challenging times. The recommendations align with a trauma-informed approach, given the high rates of death anxiety and ambiguous loss among students, and have immediate implications for educators teaching during the pandemic, and for years to come. Finally, we also advocate for more university and community-based thanatology, and gerontology education offerings in general, to help normalize conversations about death, loss, and bereavement.

